# Integrate Candidate Answer Extraction with Re-Ranking for Chinese Machine Reading Comprehension

**DOI:** 10.3390/e23030322

**Published:** 2021-03-08

**Authors:** Junjie Zeng, Xiaoya Sun, Qi Zhang, Xinmeng Li

**Affiliations:** College of Systems Engineering, National University of Defense Technology, Changsha 410073, China; zengjunjie13@nudt.edu.cn (J.Z.); zhangqiy123@nudt.edu.cn (Q.Z.); xml.nudt@gmail.com (X.L.)

**Keywords:** answer re-ranking, extraction-based machine reading comprehension, pre-training language model, self-attention

## Abstract

Machine Reading Comprehension (MRC) research concerns how to endow machines with the ability to understand given passages and answer questions, which is a challenging problem in the field of natural language processing. To solve the Chinese MRC task efficiently, this paper proposes an Improved Extraction-based Reading Comprehension method with Answer Re-ranking (IERC-AR), consisting of a candidate answer extraction module and a re-ranking module. The candidate answer extraction module uses an improved pre-training language model, RoBERTa-WWM, to generate precise word representations, which can solve the problem of polysemy and is good for capturing Chinese word-level features. The re-ranking module re-evaluates candidate answers based on a self-attention mechanism, which can improve the accuracy of predicting answers. Traditional machine-reading methods generally integrate different modules into a pipeline system, which leads to re-encoding problems and inconsistent data distribution between the training and testing phases; therefore, this paper proposes an end-to-end model architecture for IERC-AR to reasonably integrate the candidate answer extraction and re-ranking modules. The experimental results on the Les MMRC dataset show that IERC-AR outperforms state-of-the-art MRC approaches.

## 1. Introduction

Machine Reading Comprehension (MRC), one of the most difficult problems in the field of natural language processing (NLP), concerns how to endow machines with the ability to comprehend a given context paragraph and answer corresponding questions. As deep-learning technologies develop rapidly and some large-scale benchmark datasets are released, more and more researchers have proposed various end-to-end neural network models to solve this task.

Reading comprehension models can be divided into generation-based models and extraction-based models according to the way answers are generated. The former generates answers word by word, which can give neutral answers, but it lacks a global perspective, which makes it difficult to understand the answer in context; the latter extracts continuous fragments directly from the given passages, which can use the given context information to locate semantic units related to the questions [1]. Wang et al. [2] compared the performance of the generation-based and extraction-based models in its proposed Match-LSTM method. The experimental results showed that the latter is superior to the former on the Stanford Question Answering Dataset (SQuAD) v1.1. This paper focuses on how to improve the performance of extraction-based reading comprehension methods.

Word-embedding methods that are used to translate natural language texts into computer-readable mathematical vectors play an important role in extraction-based reading comprehension models. They can be roughly divided into traditional static word-embedding methods and contextualized word-embedding methods. The former such as word2vec [3], GloVe [4], can solve “the curse of dimensionality” in a one-hot representation by mapping each word to a fixed-length vector. However, it cannot effectively deal with the complex characteristics of word use or polysemy. To solve these problems, language representation models like Embeddings from Language Model (ELMo) [5], Generative Pre-Training (GPT) [6], Bidirectional Encoder Representation from Transformers (BERT) [7] and Robustly optimized BERT Pretraining approach (RoBERTa) [8] can use Bidirectional-Long Short Term Memory (Bi-LSTM) [9] or Transformer [10] models pre-trained in the large-scale corpus to generate word vectors dynamically according to context. RoBERTa [8], an improved version of BERT, has made outstanding achievements in various NLP tasks such as reading comprehension, and Named Entity Recognition, but in the Chinese reading comprehension task, the pre-training task of masked language model which randomly masks characters in the sentence and then predicts the masked characters, is poor at capturing semantic characteristics at the Chinese word level.

Answer prediction algorithms in extraction-based reading comprehension methods mainly include boundary prediction, BIO sequence prediction, word-level binary prediction and span prediction. [11]. Lee et al. [11] compared performances of different answer prediction algorithms and found that the boundary prediction algorithms that directly predict start and end indices of an answer perform the best. Therefore, extraction-based reading comprehension models proposed by follow-up research, such as Match-lstm [2], Bi-daf [12], R-net [13], Mnemonic Reader [14], all use a boundary prediction algorithm to obtain the probability distribution of the start and end indices of an answer, and then select the final answer, which has the largest joint probability of start and end indices. However, the above-mentioned reading comprehension models, where the predicted answers are only determined by the value of joint probability, are susceptible to noise data, semantic loss of word embedding and attention biases. 

To address the above-mentioned problems, this paper proposes an Improved Extraction-based Reading Comprehension method with Answer Re-ranking (IERC-AR). It consists of two modules: the candidate answers extraction module and the candidate answers re-ranking module. The former is used to construct a candidate answer set and adopts an improved pre-trained language model, RoBERTa-WWM, to extract Chinese semantics characteristic at the word level by substituting the character masking task with the whole word masking (WWM) task [7]. The latter re-evaluates candidate answers by combining the background information with the attention relationships between questions and candidate answers. The re-ranking module also constructs the self-attention network architecture to improve the accuracy of answer prediction and enhance the robustness of the model. Prior work has shown that a pipeline of these two modules can improve overall performance [15]. However, there are two major drawbacks to the pipeline system. Firstly, independently training these two modules will lead to inconsistent data distribution between the training and evaluation phase, which means that in the training phase, the input data for every module is manually constructed datasets, while in the evaluation phase, the input data for the downstream module is upstream output. Secondly, it is inefficient for each module to encode the input repeatedly. To overcome these two disadvantages, this paper designs an end-to-end model architecture to integrate answer extraction and re-ranking modules; besides, the architecture can improve overall efficiency by dealing with the problem of re-encoding.

The main contributions of this paper are as follows:

(1) The IERC-AR algorithm introduces an improved pre-trained language model, RoBERTa-WWM, which can effectively deal with complex word deformation and the problem of polysemy. And RoBERTa-WWM replaces character masking with whole word masking in the pre-training task, which can better extract semantic information at the Chinese word level.
(2) The IERC-AR algorithm uses an end-to-end architecture to integrate answer extraction and re-ranking models that can effectively solve the problem of inconsistent data distribution between the training and testing phases. In addition, the answer re-ranking module based on self-attention can highlight information related to questions in candidate answers, thereby improving the accuracy of answer prediction.
(3) This paper fully evaluates the proposed algorithm on the Les Military Machine Reading Comprehension (Les MMRC) dataset, which is a large-scale Chinese reading comprehension dataset that includes evaluations on the overall performance and effectiveness of each component.

The rest of this paper is organized as follows: Section 2 discusses some related work on reading comprehension methods, word embedding methods and answer re-ranking methods; Section 3 presents the proposed approach; Section 4, evaluates the approach’s performance in the Les Military Machine Reading Comprehension dataset; and Section 5 offers conclusions.

## 2. Related Work

This section will introduce related works regarding contextualized word-embedding methods, answer re-ranking methods and reading comprehension methods. 

### 2.1. Contextualized Word Embedding Methods

Traditional word embedding methods such as word2vec and GloVe, can convert natural language texts to word vectors with fixed length, and solve “the curse of dimensionality” in one-hot representation. However, it is difficult for above methods to handle various vocabulary deformations and the problem of polysemy. To address above problems, researchers have proposed contextualized word embedding methods based on pre-trained language models, which make significant progress in many natural language processing tasks such as reading comprehension, text classification and named entity recognition.

The core idea of word embedding methods based on the pre-trained language model is that the language model is firstly pre-trained in a large-scale unsupervised corpus and then used to build word vectors with semantic information for the input texts in downstream target tasks. These methods can be divided into two types according to the training method: the autoregressive language model or the self-coding language model. Several state-of-the-art pre-trained language models such as ELMo [5], GPT [6], BERT [7], RoBERTa [8] are described in detail below.

ELMo [5] adopts a bidirectional LSTM language model, which is composed of a forward and backward language model. First, the LSTM network is used to learn the complex features of word deformations and then join the forward and backward language model’s feature representation. If there are multiple layers of LSTM in the model, each layer has its own weight, and the weight can be learned from the training process of downstream tasks. Compared with ELMo, GPT [6] adopts the transformer-based neural network, which can better capture long-range dependency relationships, but it can only predict the word according to the text before the word. 

BERT [7] adopts a bidirectional Transformer-based architecture and uses the masked language model and next sentence prediction tasks to capture semantic features. In general, the bidirectional Transformer language model adopted by BERT can not only efficiently capture long-range dependencies but also capture bidirectional context information. However, the use of masking operations in the pre-training stage would cause a mismatch in the fine-tuning phase, which would influence the performance of the model in downstream tasks.

RoBERTa [8] optimizes the pre-training method from four aspects: (1) replacing static masking with dynamic masking, which means the masked tokens it chooses will change during the training progress; (2) removing the next sentence prediction task; (3) using a larger batch size for pre-training; (4) and using more pre-training data and longer pre-training time.

Although RoBERTa improves performances by optimizing the pre-training method, the MLM task is not suitable for Chinese dataset. Chinese pays more attention to the semantic information at the word level, while character masking in the MLM task is not suitable to capture the word level semantics. RoBERTa-WWM [16] substitutes character masking with WWM in the MLM task, thereby improving the extraction of Chinese word-level semantic features.

### 2.2. Answer Re-Ranking Methods

In the field of answer re-ranking, Jansen et al. [17] built answer re-ranking models based on grammar, lexical semantics and discourse, which could use discourse information to supplement distributed lexical semantic information. To improve Jansen’s lexical semantic model, Fried et al. [18] used a more advanced model to take advantage of indirect connections between words. Then, with the popularity of community-based Q&A sites, such as Yahoo! Answers and Quora, a large amount of precious training datasets were accumulated. Bogdanova et al. [19] did not continue to study the answer-ranking model from the perspective of vocabulary semantics but used the neural-network method to predict the answers by a fully connected network and achieved better results compared with former models.

However, those methods do not consider background knowledge to match the best answer for the question. In order to solve the problem of re-ranking answers with background information, this paper proposes an answer re-ranking model based on self-attention. To improve the accuracy of answer re-ranking, the proposed model makes full use of the background and question information to generate an answer representation through the attention mechanism. In addition, the traditional answer re-ranking model uses a pipeline model to cascade with the answer extraction module, which leads to mismatches between the training and testing phases, and the re-encoding for input is quite time-consuming. To solve these problems, the paper proposes to use an end-to-end architecture to integrate the candidate answer extraction module and re-ranking module.

### 2.3. Reading Comprehension Methods

Reading comprehension methods can be divided into generation-based and extraction-based methods according to the how answers are generated. Most generation-based methods, such as GTTP [20], are based on the sequence-to-sequence architecture and attention mechanism, which can generate natural, fluent answers. However, such methods tend to generate non-informative answers such as “Thank you” and “I have no idea”. To generate more informative answers, researches introduced external knowledge [21] or adjusted the objective function [22].

The extraction-based reading comprehension method extracts a continuous fragment from the given context paragraph as answers. Its model architecture comprises the representation layer, the encoding layer, the matching layer, and the extraction layer. Taking the natural language text as an input, the representation layer can generate word vectors by the word-embedding model. The encoding layer can encode the word vectors with rich semantic information by using a convolutional neural network, a recurrent neural network or even a transformer neural network. The matching layer can integrate the encoded feature vectors of the question and passage and then obtain a question-aware attention passage representation; The extraction layer outputs the probability distribution of the start and end indices of the answer. Since Pointer Net [23] only considers the prior knowledge that the output can select from the input and designs the seq2seq and attention mechanism architecture, which is very suitable for addressing the text extraction question, Wang et al. [2] combined Match-LSTM with it to predict an answer’s start and end positions. To improve the interaction between the question and passage information in the matching layer of Match-LSTM, Wang et al. proposed R-net [13] by adding the gate network, self-attention and a smooth layer. Since Both Match-LSTM and R-net use a one-way attention mechanism in the matching layer to improve the effects of attention, Seo et al. proposed Bi-daf [12] by introducing a bidirectional attention mechanism. Inspired by the human reading behavior of repeatedly reading a given passages when answering complex questions, Hu et al. [14] proposed Mnemonic Reader (M-Reader), which introduced an iterative mechanism in the matching layer to improve inference ability.

Since the above-mentioned extraction-based reading comprehension methods directly choose the predicted answer with the largest joint probability, they may be susceptible to noise data, missing semantic information of word embedding and attention biases. To solve these problems, the proposed IERC-AR algorithm adds the answer re-ranking module to re-evaluate candidate answers, which can also enhance model’s robustness and improve the accuracy of answer predictions.

## 3. Methodology

This section firstly introduces the formal description of the problem, then describes the overall architecture of the proposed algorithm, and finally describes the candidate answer extraction module and candidate answer re-ranking module, respectively.

### 3.1. Problem Description

The problem of machine reading comprehension is to predict the answer based on the given passage and the corresponding question. In other words, the problem is to predict an answer $A=\left\{a_{1},a_{2},\cdots ,a_{k}\right\}A=\left\{a_{1},a_{2},\cdots ,a_{k}\right\}$ in a given passage $P=\left\{p_{1},p_{2},\cdots ,p_{n}\right\}$ and question $Q=\left\{q_{1},q_{2},\cdots ,q_{m}\right\}$, as shown in Equation (1),

(1) A=f(P,Q)

where f() represents the trained model. 

This paper focuses on the extraction-based reading comprehension method where answer A is a continuous segment of the passage. Specifically, the problem is to predict the start and end indices of the answer in the passage, as shown in Equation (2),

(2) $$\begin{array}{c} \left(s_{p},e_{p}\right)=f\left(P,Q\right)\\ a=P_{\left(s_{p},e_{p}\right)} \end{array}$$

where $s_{p}$ is the start index and $e_{p}$ is the end index.

### 3.2. Algorithm Architecture

**Figure 1:**
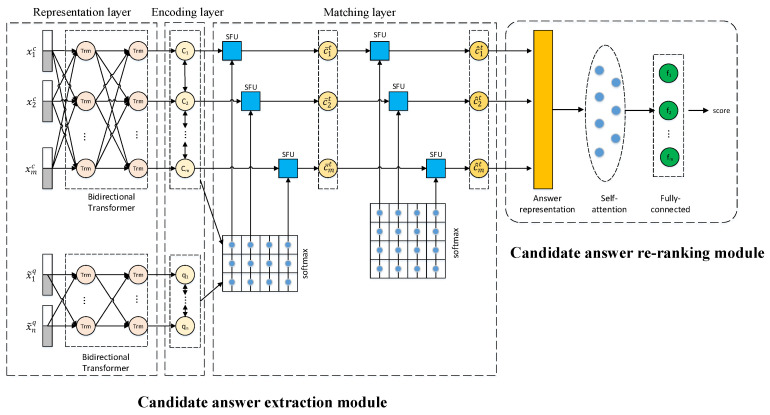
The overall architecture of IERC-AR.

This paper proposes an improved extractive reading comprehension algorithm, named IERC–AR, to combine the answer extraction module with the answer re-ranking module end-to-end. The model framework is shown in Figure 1. The model consists of two modules, namely, the answer extraction module and answer re-ranking module. To solve the mismatch between these two modules during the training and testing phases and the problem of re-encoding for input, IERC-AR proposes an end-to-end model architecture to integrate answer extraction and re-ranking modules. In order to solve the problem of polysemy and capture precise semantic features at the Chinese word-level, the candidate answer extraction module adopts RoBERTa-WWM to generate word vectors. To improve the accuracy of answer prediction, the answer re-ranking module uses a self-attention model to re-evaluate candidate answers.

The proposed algorithm has the following three advantages:

(1) The IERC-AR algorithm proposes an end-to-end architecture to integrate the answer extraction module and the answer re-ranking module. The traditional answer re-ranking methods mostly cascade different modules into a pipeline, leading to a mismatch between these two modules during the training and testing phases. For example, the model proposed by Wang et al. [24] consists of retriever, reader and re-ranking modules in a pipelined way, and Wang et al. [25] integrated answer extraction, re-ranking and other modules by constructing a pipeline system. The proposed end-to-end architecture uses the output of the answer extraction module as the input of the re-ranking module in both the training and testing phases, so the mismatch problem can be effectively avoided. In addition, the answer re-ranking module and other modules need to encode the input texts individually, causing a waste of computation resources. The proposed end-to-end architecture can effectively solve the problem of re-encoding by sharing the representation layer, encoding layer and matching layer of the answer extraction modules with the answer re-ranking module.
(2) The answer extraction module uses the improved pre-trained language model RoBERTa-WWM, to dynamically generate word vectors by considering the context, which can solve the polysemy problem. Firstly, the bidirectional Transformer architecture used in RoBERTa-WWM can efficiently capture long-range dependencies to generate informative word representations for the input texts. Secondly, RoBERTa-WWM replaces character masking with WWM in the pre-training task, which is helpful for extracting semantic features at the Chinese word level.
(3) To improve the accuracy of evaluating candidate answers, the answer re-ranking module not only adds background information but also designs a self-attention mechanism. The traditional answer re-ranking method only measures semantic relationships between candidate answers and questions, without considering the background information. The answer re-ranking module in IERC-AR can combine the context with the question to the word representation of the candidate answer containing accurate semantic information. In addition, the self-attention mechanism used in the answer re-ranking module can capture long-range interdependence. This module takes the answer representation with question information as the input of the self-attention layer, which can further fit the answer representation, highlight key information and reduce the interference of invalid information, thus improving the overall performance of the answer re-ranking module.

### 3.3. Candidate Answer Extraction Module

#### 3.3.1. Representation Layer

The representation layer of the answer extraction module of the IERC-AR algorithm uses the improved pre-trained language model—RoBERTa-WWM to dynamically generate word vectors. RoBERTa-WWM uses the bidirectional Transformer as an encoder. Compared with the RNN architecture, Transformer adopts self-attention structure, which can directly obtain global information, support parallel computing and better handle long-range dependencies. In addition, the bidirectional Transformer architecture can generate the word vector with accurate semantic information according to the context. 

**Figure 2:**
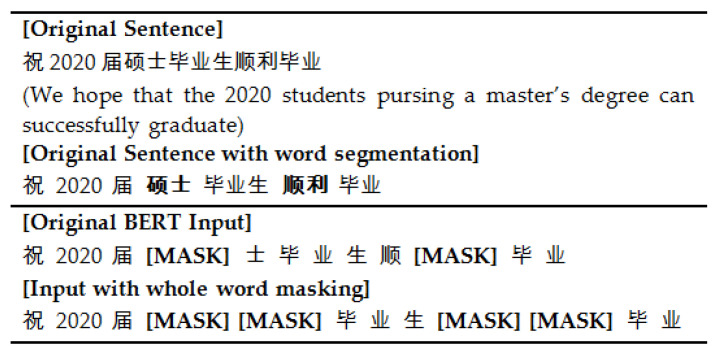
Example of whole word masking in RoBERTa-WWM.

Compared with the BERT model, RoBERTa-WWM improves its pre-training methods in three aspects:

(1) It replaces character masking with WWM in the masked language model. The character used in BERT first applies the word segmentation method to divide a word into several characters, and then randomly masks these separated characters. For WWM used in RoBERTa-WMM, some characters of the word are masked but others belonging to the same word will also be masked, as shown in Figure 2. The pre-training task of the masked language model based on WWM is helpful for capturing semantic features at the Chinese word level, thereby improving the overall performance of the model.
(2) It substitutes static masking with dynamic masking in the masked language model task. The static masking of BERT is randomly selects 15% of the tokens for each sequence and replace them with [MASK], and the masked tokens will not change during the pre-training process. In the dynamic masking used in RoBERTa-WMM, the masked word is newly chosen tokens in each iteration epoch.
(3) It removes the next-sentence prediction task during the pre-training phase. In BERT pre-training, the positive samples are selected from the same document and the negative samples are chosen in documents with different topics, so it is quite easy for the neural network model to distinguish them.

RoBERTa-WWM uses a large-scale Chinese corpus for pre-training, such as Chinese Wikipedia, encyclopedia, news and Q&A. More information about pre-training can be found in https://github.com/ymcui/Chinese-BERT-wwm (accessed on 16 April 2020). When the pre-training stage finishes, RoBERTa-WWM can be applied to machine reading comprehension in feature-based or fine-tuning based manner. Fine-tuning based is the mainstream way to apply pre-trained language models. This method connects the pre-trained language model with a fully connected layer, which can be fine-tuned in a short time to adapt to the downstream task. However, in the Chinese machine reading comprehension task, due to the need for efficient parallel processing of large-scale datasets in the pre-training stage, the model structure corresponding to fine-tuning based is simple and cannot generate enough precise question-aware attention passage representation. Therefore, this paper chooses the feature-based method of using the RoBERTa-WWM model. This paper only uses the pre-trained model to generate word vectors in the representation layer, and the parameters of the pre-trained model are kept unchanged in the training process.

In the representation layer, WordPiece is used to segment the question and the original text. Because the given passages in the dataset are too long to be input directly, the key sentences need to be extracted first. The details of key sentence extraction will be described in detail in the data preprocessing section of Section 4.3.

After the word segment, separators are added to the questions at the beginning and end position, and then the input sequence is obtained. The same operators are executed in the passages. Take the input sequence of the question as an example: $Q_{sep}=\left[\left[CLS\right],\;q_{2},\ldots ,\;q_{l_{q}-1},\;\left[SEP\right]\right]$, where [CLS] denotes a classification label; [SEP] denotes separation label; and $l_{q}$ denotes the length of the sequence. The feature representations of each element in the input sequence include three parts: word index, sentence label and mask label. The language model takes feature representations of the passage and question as inputs, and dynamically generates word vectors respectively according to the context. Then, the bidirectional LSTM layer is used to encode word vectors of passages and questions, respectively.

#### 3.3.2. Matching Layer

The matching layer uses an iterative mechanism to enhance interactions between questions and passages. Each iteration block is composed of three parts: interactive aligning, self-aligning and aggregating, which are described as follows.

Interactive aligning involves constructing matching relations between passages and questions as estimated by the inner product. The matching relation matrix can be used to calculate a new question representation vector, shown in Equation (3),

(3) $$\begin{array}{c} D_{ij}^{t}=q_{i}^{T}*\bar{c_{j}^{t-1}}\\ \bar{c_{j}^{0}}=c_{j}\\ d_{j}^{t}=softmax\left(D_{:j}^{t}\right)\\ \bar{q_{j}^{t}}=Q*d_{j}^{t} \end{array}$$

where D denotes a matching relation matrix; $D_{ij}^{t}$ denotes the similarity between the $i_{th}$ word in a question and the $j_{th}$ word in a passage; $d_{j}^{t}$ denotes an attention distribution of a question with respect to a passage; $\bar{q_{j}^{t}}$ denotes a question representation vector based on attention of a passage; $\bar{c_{j}^{t-1}}$ denotes a passage representation vector. 

Based on $\bar{c_{j}^{t-1}}$ and $\bar{q_{j}^{t}}$, the question-aware passage representation $\overset{=}{c_{j}^{t}}$ can be calculated by the Semantic Fusion Unit (SFU) as shown in Equation (4).

(4) $$\overset{=}{c_{j}^{t}}=SFU\left(\bar{c_{j}^{t-1}},\;\bar{q_{j}^{t}},\bar{c_{j}^{t-1}}*\bar{q_{j}^{t}},\;\bar{c_{j}^{t-1}}-\bar{q_{j}^{t}}\right)$$

The SFU can fuse question information into passage representation as shown in Equation (5),

(5) $$\begin{array}{c} \bar{r}=\tan h\left(W_{r}\left(\left[r;f_{1},\ldots ,f_{k}\right]\right)+b_{r}\right)\\ g=\sigma \left(W_{g}\left(\left[r;f_{1},\ldots ,f_{k}\right]\right)+b_{g}\right)\\ o=g*\bar{r}+\left(1-g\right)*r \end{array}$$

where r denotes main information, and $\left[f_{1},\ldots ,f_{k}\right]$ denotes merged information.

The part of self-aligning can enhance understanding of long sequence texts by strengthening important information. Self-aligning is similar to interactive aligning, but the main difference between them is how to calculate the matching relation matrix. Interactive aligning calculates the matching relation between P and Q, while self-aligning calculates the matching relation between P and P, shown in Equation (6),

(6) $${\bar{D}}_{ij}^{t}=J_{\left(i\neq j\right)}{\overset{=}{c_{j}^{t}}}^{T}*\overset{=}{c_{j}^{t}}$$

where $J_{\left(\cdot \right)}$ is an indicator function used to ensure that the contexts are not aligned with themselves.

The part of aggregating adopts the recurrent neural network to further fit the passage representation and then calculates fitted passage representation $\overset{↔}{c}$.

#### 3.3.3. Extraction Layer

The extraction layer adopts a pointer network with memory. And this network maintains a memory vector $z_{s}^{l}$ that used to record necessary information when predicting answers. The initial state of $z_{s}^{l}$ is final state of the encoded question. Assuming that the number of iterations of the pointer network contains is L, at the $l_{th}$ iteration the probability distribution of start indices of the answer is shown in Equation (7),

(7) $$\begin{array}{c} s_{j}^{l}=FN\left({\overset{↔}{c_{j}}}^{T},z_{s}^{l},{\overset{↔}{c_{j}}}^{T}*z_{s}^{l}\right)\\ p_{s}^{l}\left(j\right)=softmax\left(w_{s}^{l}*s_{j}^{l}\right) \end{array}$$

where $p_{s}^{l}\left(j\right)$ denotes the probability distribution of an answer’s start index, and FN denotes fully connected neural network used to fit data in nonlinear way.

The evidence vector $u_{s}^{l}={\overset{↔}{c}}^{T}*p_{s}^{l}$ can be used to update $z_{s}^{l}$. The expression of update is $z_{e}^{l}=SFU\left(z_{s}^{l},u_{s}^{l}\right)$, where SFU is described in Equation (5). Similar to the calculation of $p_{s}^{l}\left(j\right)$, the probability distribution of the end indices of the answer is shown in Equation (8).

(8) $$\begin{array}{c} e_{i}^{l}=FN\left({\overset{↔}{c_{i}}}^{T},z_{e}^{l},{\overset{↔}{c_{i}}}^{T}*z_{e}^{l}\right)\\ p_{e}^{l}\left(i\right)=softmax\left(w_{e}^{l}e_{i}^{l}\right) \end{array}$$

To build the candidate answer set, the answer extraction module in IERC-AR selects candidate answers according to the value of joint probability. The size of the candidate answer set $count_{ans}$ is 5. Assuming that the probability of the answer’s start index and end index are independent, the joint probability of start and end indices of the answer is shown in Equation (9),

(9) $$\mathbb{P}\left({\mathrm{answer}}_{pos_{s}},{\mathrm{answer}}_{pos_{e}}\right)=\mathbb{P}\left({\mathrm{answer}}_{pos_{s}}\right)*\mathbb{P}\left({\mathrm{answer}}_{pos_{e}}\right)$$

where ${\mathrm{score}}_{ex}$ is the score of the candidate answer evaluated by the answer extraction module, described in Equation (10).

(10) $${\mathrm{score}}_{ex}=\mathbb{P}\left({\mathrm{answer}}_{pos_{s}}\right)+\mathbb{P}\left({\mathrm{answer}}_{pos_{e}}\right)$$

### 3.4. Candidate Answer Re-Ranking Module

Since a pipeline of the answer extraction module and re-ranking module leads to the re-encoding problem and inconsistent data distribution between training and testing phases, IERC-AR proposes a novel end-to-end architecture to integrate different modules. In this architecture, the answer extraction module shares the representation layer, encoding layer and matching layer with the answer re-ranking module. Therefore, the re-ranking module can use RoBERTa-WWM to gain the precise answer representation by considering the context, and obtain the question-aware answer representation by the matching layer.

The self-attention layer in the answer re-ranking module takes the question-aware answer representation as the input, and then calculates the attention distribution between interior elements, which can capture relations among words in the answer and generate the precise answer representation. The output of the self-attention layer successively passes through a fully connected layer, a dropout layer and a linear layer to grade candidate answers.

To train the candidate answer re-ranking module, we constructed two kinds of labels for each candidate answer $c\_ans_{i}$. These labels are the hard label $y^{hard_{i}}$ and the soft label $y^{soft_{i}}$. The value of $y^{hard}$ is binary, which determines if $c\_ans_{i}$ is the same as the reference answer or not. The value range of $y^{soft}$ is [0, 1], which denotes the Recall-Oriented Understudy for Gisting Evaluation-L (ROUGE-L) score between the candidate answer and the reference answer. Since in the early training phase there are only a few positive samples in the candidate answer set generated by the answer extraction module, making it difficult to train the answer re-ranking module efficiently, we substitute the candidate answer with the lowest value of $y^{soft}$ with the reference answer when there is no positive sample. 

The output of the answer re-ranking module, ${\mathrm{score}}_{re}$, denotes the score of the candidate answer given by the answer re-ranking module.

The final score of candidate answer synthesizes the scores of the answer extraction module and re-ranking module, shown in Equation (11).

(11) $$score=score_{ex}+\gamma *score_{re}$$

where γ is a factor used to control the weight of the answer re-ranking module’s evaluation.

## 4. Experiment

### 4.1. Dataset

The Dataset adopted by this paper is Les Military Machine Reading Comprehension released by China Electronic Technology Les Corporation, called Les MMRC for short. Les MMRC is the large-scale dataset in the field of Chinese machine reading comprehension. It contains 40,000 passages and 200,000 question-answer pairs, which makes it an excellent platform for training and testing models. This paper divides the whole dataset into a training set and a testing set, the proportion of which is 9:1.

This dataset contains six kinds of questions: passage-based, definition-based, list-based, fact-based, number-based and opinion-based. The passage-based questions focus on inferences about several paragraphs. The definition-based questions pay attention to definitions of terms. The form of answers of list-based questions is the list. The fact-based questions involve how to interpret certain events described in the passage. In the number-based questions the answer format is a numerical value. The opinion-based questions entail clarifying viewpoints about an event in the passage. One sample in Les MMRC is shown in Table 1.

### 4.2. Evaluation Indicator

This paper adopts Bilingual Evaluation Understudy (BLEU) and ROUGE-L as evaluation indicators for performance on machine reading comprehension models. The two indicators are widely used in NLP tasks like machine translation and machine reading comprehension.

BLEU, proposed by Kishore Papineni et al. [26], is a well-known evaluation indicator in NLP that can calculate scores by comparing candidate texts generated by models with one or multiple reference texts. Specifically, this indicator firstly counts n-gram in candidate texts and reference texts, and then compares the number of their n-grams. The range of BLEU is [0, 1], and when the candidate texts and reference texts are exactly matched, the score of BLEU is 1. The calculation of BLEU is shown in Equation (12).

(12) $$BLUE=BP*e^{\left[\left(\overset{N}{\underset{n=1}{\sum }}w_{n}\log P_{n}\right)\right]}$$

where $w_{n}$ denotes the weights of co-occurrence matrix of n-gram. This paper calculates BLEU by the number from 1-g to 4-g, and $w_{n}=\left[0.25,\;0.25,0.25,0.25\right]$. Reference [26] has proved that when the calculation of BLEU depends on 1-gram to 4-gram, its results are similar to a human evaluation result. Brevity Penalty (BP) is shown in Equation (13).

(13) $$\mathrm{BP}=\{\begin{array}{c} 1\\ e^{\left(1-count_{r}/count_{c}\right)} \end{array}\begin{array}{c} if\;count_{c}>\;count_{r}\\ if\;count_{c}\leq count_{r} \end{array}$$

where $count_{r}$ and $count_{c}$ are the number of n-g reference texts and candidates texts, respectively.

$P_{n}$ denotes the accuracy of N-gram, shown in Equation (14).

(14) $$P_{n}=\frac{\sum_{C\in candidates}\sum_{n-gram\in C}Count_{clip}\left(n-gram\right)}{\sum_{C\in candidates}\sum_{n-gram\in C}Count\left(n-gram\right)}$$

where $Count_{clip}\left(n-gram\right)$ is the final number of n-g, shown in Equation (15).

(15) $$Count_{clip}\left(n-gram\right)=min\left(Count\left(n-gram\right),\;MaxRefCount\left(n-gram\right)\right)$$

where Count(n−gram) is the occurrence number of n-g in candidate texts, and MaxRefCount(n−gram) is the max occurrence number of n-gram in reference texts.

Recall-Oriented Understudy for Gisting Evaluation (ROUGE) [27] is a kind of similarity matric based on recall rate. Similar to BLEU, it mainly investigates the sufficiency and loyalty of candidate texts by calculating the co-occurrence frequency of n-gram between reference texts and candidate texts. ROUGE has four categories: ROUGE-N, ROUGE-L, ROUGE-W and ROUGE-S.

This paper uses ROUGE-L as the evaluation indicator, where L denotes the Longest Common Subsequence (LCS), shown as in Equation (16),

(16) $$\begin{array}{c} R_{lcs}=\frac{LCS\left(X,Y\right)}{l_{m}}\\ P_{lcs}=\frac{LCS\left(X,Y\right)}{l_{n}}\\ F_{lcs}=\frac{\left(1+\beta^{2}\right)R_{lcs}*P_{lcs}}{R_{lcs}+\beta^{2}*P_{lcs}} \end{array}$$

where LCS(X, Y) is the length of the longest common subsequence among X and Y; $l_{m}$ and $l_{n}$ denote the lengths of reference texts and candidate texts, respectively; $R_{lcs}$ denotes the recall rate, while $P_{lcs}$ denotes the accuracy rate; $F_{lcs}$ is the score of ROUGE-L; β is generally set as a large number so that ROUGE-L merely considers $R_{lcs}$.

### 4.3. Data Preprocessing

Since a lot of data are noisy, redundant or incomplete in the Les MMRC dataset, we cleaned it by the way of data preprocessing, which consists of three parts: data conversion and filtering, core texts extraction and answers labelling. 

In the data conversion and filtering,

(a) all Chinese traditional characters are converted to simplified characters;
(b) all full-width numbers or letters are converted to half-width;
(c) extra punctuation at the beginning or end of answers is deleted;
(d) space characters like ‘\u8000′ and ‘\t’ are converted to blank spaces;
(e) a blank space at the beginning or end of a title, passage, question or answer are deleted.
(f) false data like an identical question or a title with the answer are deleted; and
(g) data with no answer are deleted.

In the core texts extraction, some passages are very long, which brings great computational cost to the training phase, so it is important to extract core texts. Before extracting the texts, we successively use jieba and WordPiece to do word segmentation. Then, we obtained the sentence list by sentence segmentation and extracted sentences by the designed rules until the length reached the given threshold. The rules contain the priorities of different sentences, which are described as follows:

(1) title;
(2) the core sentence whose has the highest ROUGE-L value with a question;
(3) the next sentence of the core sentence
(4) the last sentence
(5) the first sentence
(6) the sentence containing the subsequence of the question, its previous and following sentences
(7) the second sentence after the core sentence
(8) the sentence before the core sentence
(9) the third sentence after the core sentence
(10) the second sentence before the core sentence

When reduplicated sentences appear in the extracted sentence list, only one will remain. The extraction is accurate only if the answer appears in the extracted text. This article only uses accurate samples as training samples. The maximum length of the extracted text is 500.

Since many answers appear more than once in the given passage, when answer labelling we label the most suitable answer by calculating ROUGE-L between answers in context and the question.

### 4.4. Baselines

This paper chose six methods widely used in the field of machine reading comprehension as baselines: RoBERTa based on Fine-Tuning [8], Match–LSTM [2], R-net [13], Bi-daf [12], M_Reader [14] and QA-net [28], which are trained in the Les MMRC dataset.

RoBERTa based on Fine-Tuning [8] adds an output layer, like a simple, fully connected layer, to RoBERTa, a state-of-the-art pre-training language model, and fine tunes parameters according to downstream NLP tasks. This paper uses the pointer network as the output layer. The setting of hyper-parameters is as follows: Learning rate is 4 × 10^−5^; Batch size is 32; In the pointer network, the number of hidden units is 384, and the value of dropout is 0.25; Other hyper-parameters are identical with reference [8].

The external word2vec, trained by various kinds of corpus, is used as the word embedding model in other baselines. These baselines, except RoBERTa based on Fine-Tuning, adopt bidirectional LSTM or bidirectional Gated Recurrent Unit (GRU) in the encoding layer. We do not change structures of the matching and extraction layer in the other five kinds of baselines, which are implemented according to original papers. The setting of the hyper-parameters of other baselines is as follows: the learning rate is 10^−4^; the batch size of Match–LSTM and QA-net are 96 and 12 respectively; and the batch size of the other methods is 128. In the encoding layer, the number of hidden units of Match–LSTM and QA-net are 150 and 128 respectively, and in the other methods, it is 100; The value of dropout in the encoding layer is 0.1.

### 4.5. Settings

The loss function of IERC-AR is shown in Equation (17).

(17) $$Loss_{QA-RCA}=Loss_{ex}+Loss_{re}$$

where $Loss_{ex}$ denotes the loss function of the candidate answer extraction module, and $Loss_{re}$ denotes the candidate answer re-ranking module’s loss function. 

$Loss_{ex}$ is the maximum likelihood loss function, shown in Equation (18).

(18) $$Loss_{ex}=-\frac{1}{l_{p}}\overset{l_{p}}{\underset{i=1}{\sum }}\left(NLLLoss(\log y_{s},label_{s})\;+NLLLoss(\log y_{e},label_{e})\right)$$

The candidate answer re-ranking module adopts two kinds of labels: the hard label $y^{hard}$ and the soft label $y^{soft}$. The value of $y^{hard}$ is binary. $y^{hard}$ equals 1, meaning it is exactly matched between the candidate answer and the reference answer. $y^{soft}$ denotes the value of ROUGE-L between the candidate answer and the reference answer. Different labels have corresponding loss functions. For $y^{hard}$, we adopted the cross-entropy loss function that is well-received in binary classification or multi-classification problems, as shown in Equation (19).

(19) $$Loss_{re}^{hard}=-\overset{count_{ans}}{\underset{i=1}{\sum }}y^{hard_{i}}\log\left(softmax\left({\mathrm{score}}_{re}{}_{i}\right)\right)$$

For $y^{soft}$, we adopted the Mean-Square Error (MSE) loss function, described in Equation (20).

(20) $$Loss_{re}^{soft}=\frac{1}{count_{ans}}\overset{count_{ans}}{\underset{i=1}{\sum }}{||y^{soft_{i}}-score_{re_{i}}||}^{2}$$

The loss function of the candidate answer re-ranking module is the weighted sum of $Loss_{re}^{hard}$ and $Loss_{re}^{soft}$, as shown in Equation (21),

(21) $$Loss_{re}=\alpha *Loss_{re}^{hard}+\beta *Loss_{re}^{soft}$$

where α and β are parameters that are used to control the weights of $Loss_{ex}$ and $Loss_{re}$, shown in Equation (22),

(22) $$\begin{array}{c} \{\begin{array}{c} \alpha =0.1\\ \beta =10 \end{array}\;if\;epoch_{t}\leq 4\\ \{\begin{array}{c} \alpha =0\\ \beta =20 \end{array}\;if\;epoch_{t}>4 \end{array}$$

where $epoch_{t}$ denotes the iteration round at time t. In Equation (22), α and β change as the iteration round changes, and $Loss_{re}$ is called the stage-loss function. At the beginning of training, since the candidate answer set generated by the poor candidate answer extraction module is of low quality, adding $Loss_{re}^{hard}$ to $Loss_{re}$ can speed up the process of convergence. However, there are some contradictions between $Loss_{re}^{hard}$ and $Loss_{re}^{soft}$. Specifically, $Loss_{re}^{hard}$ aims to increase the score of right candidate answers and decrease the score of wrong answers, while $Loss_{re}^{soft}$ aims to make the score of candidate answers approach its ROUGE-L score. Therefore, in the middle and late stages of training, considering that the answer extraction module can generate the high-quality candidate answer set, we removed the $Loss_{re}^{hard}$ and increased the weight of $Loss_{re}^{soft}$ in the loss function $Loss_{re}$. This paper discusses the impact of weights α and β on the performance of the answer re-ranking module in Section 4.7.

Considering that the number of training epochs required to converge is different among various methods, the epochs of IERC-AR and baselines except RoBERTa based on Fine-Tuning are set at 20, and this paper adopted the strategy of early stopping to avoid overfitting. We set the training epoch of RoBERTa based on Fine-Tuning at 4.

The hyper-parameters of IERC-AR are set as follows: Learning rate is 10^−4^; batch size is 256; the maximum of gradient descent is 5; the hyper-parameters of RoBERTa-WWM is identical to RoBERTa based on Fine-Tuning in Section 4.4; the encoding layer consists of bidirectional LSTM, whose number of hidden units is 100, and the value of dropout is 0.1; In the matching layer, the number of hidden units is 200, and the value of dropout is 0.25; In the extraction layer, the number of hidden units is 200, and the value of dropout is 0.25; In the candidate answer re-ranking module, the number of hidden units is 200, and the value of dropout is 0.1.

In the experimental platform used in this paper, the operating system is 64-bit Ubuntu 16.04 or high. The CPU is Inter (R) Xeon (R) CPU E5-2678 v3 @ 2.50 GHz, while the GPU is 4 RTX 2080Ti. The memory size is 128 G, and the deep learning framework is pytorch 1.0 released by Facebook.

### 4.6. Experimental Results and Analysis

#### 4.6.1. Evaluation on the Performance of IERC-AR

**Figure 3:**
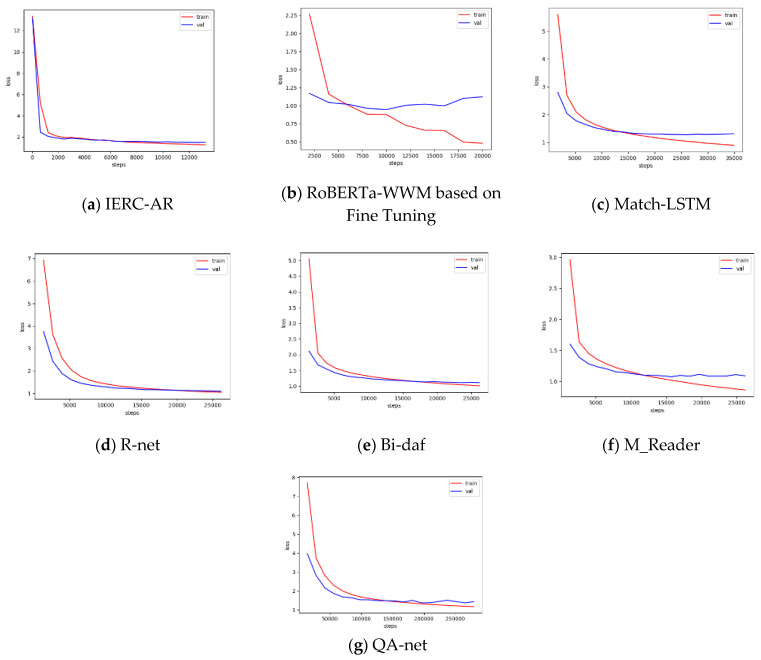
Loss values curve of IERC-AR and baselines in the training phase.

In Figure 3, the red line denotes changes of loss value in the training set, while the blue line denotes changes of loss value in the validation set. Figure 3 describes the changes of loss value of IERC-AR and baselines in the training phase, where the curves of IERC-AR, R-net and Bi-daf descend steadily at the end of the training process, and there is no obvious overfitting situation. Table 2 describes the performances of IERC-AR and baselines on Les MMRC dataset. As seen in Table 2, the scores of BLEU and ROUGE-L of IERC-AR are higher than baseline scores. As for BLEU, the score of IERC-AR is 7.79% higher than Match–LSTM, which performed the worst among baselines, and is 2.68% higher than R-net, which performed the best among baselines. Regarding ROUGE-L, the score of IERC-AR is 16.12% higher than RoBERTa-WWM based on Fine-Tuning and 1.33% higher than R-net. These experimental results show that IERC-AR not only generated the word embedding representation with more precise semantic information by using the improved pre-training language model RoBERTa-WWM, but also that it effectively integrated the candidate answer extraction module with the candidate answer re-ranking module based on self-attention mechanism by proposing a novel end-to-end architecture, which can predict answers more precisely and increase the model’s robustness.

#### 4.6.2. Evaluation of Effectiveness of IERC-AR Components

Table 3 shows that the performances of IERC-AR are better than without re-ranking module, and especially for the BLEU score, IERC-AR is 1.8% higher. This results illustrate that the answer re-ranking module can not only use the self-attention mechanism to highlight important information, but also fully utilize answer representation with background and question information shared by the candidate answer extraction module; besides, the end-to-end architecture proposed by IERC-AR can effectively integrate the answer extraction module and the answer re-ranking module.

**Figure 4:**
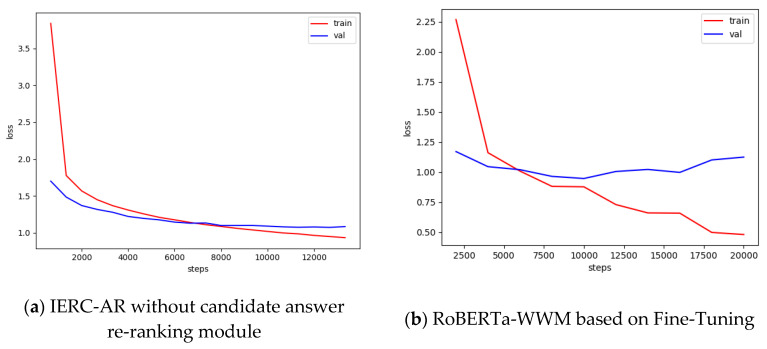
Loss values curve of IERC-AR without the candidate answer re-ranking module and RoBERTa-WWM based on Fine-Tuning in the training phase.

In Figure 4, although compared with IERC-AR without the answer re-rankng module, RoBERTa-WWM based on Fine-Tuning can converge faster, it encounters serious overfitting. As seen in the Table 4, as for BLEU, IERC-AR without the answer re-ranking module is 4.2% higher than RoBERTa-WWM based on Fine-Tuning, and for the ROUGE-L score, IERC-AR is 15.65% higher. This experimental results illustrate that although RoBERTa-WWM based on Fine-Tuning that are widely used to solve various kinds of natural language tasks can fully utilize bidirectional Transformer to extract abstract semantics representation, its performances are worse than IERC-AR without answer re-ranking module that adopts feature-based RoBERTa-WWM. To utilize powerful computation to parallel pre-train the language model in the large-scale dataset, RoBERTa based on Fine-Tuning simplifies its network architecture and does not have a specialized matching layer to merge the question representation with the passage representation. The result is that its performance on downstream tasks are worse than that of IERC-AR without the answer re-ranking module.

The main difference between IERC-AR without the candidate answer re-ranking module and M_Reader is the word embedding model used in the representation layer: the former adopts the improved pre-training language model RoBERTa-WWM, while the latter uses the traditional word embedding model word2vec. In the Table 4, scores of BLEU and ROUGE-L of IERC-AR without re-ranking module were higher than those of M_Reader. Significantly, its BLEU score was 1.71% higher than that of M_Reader. The experimental results show that, compared with word2vec, RoBERTa-WWM can utilize the bidirectional Transformer to build a contextualized word embedding model that can effectively address the problem of polysemy and generate precise word representations.

As shown in Table 4, the scores of BLEU and ROUGE-L of the method based on BERT-WWM were higher than the one based on BERT, and its BLEU score of 5.64%. was especially higher than that of BERT: These experimental results show that IERC-AR can enhance the ability to extract semantic features at Chinese word-level by substituting character masking with WWM in the pre-training phase of the language model, which can increase the accuracy of word representation.

### 4.7. Discussion

#### 4.7.1. Loss Functions of the Candidate Answer Re-Ranking Module

The loss function plays an important role in performances of the network model. In IERC-AR, $Loss_{re}$ that is loss function of candidate answer re-ranking module consists of $Loss_{re}^{hard}$ and $Loss_{re}^{soft}$. This section analyzes the influences of weights α and β on performances of the model by comparing four kinds of different loss function: $Loss_{re}^{hard}$, $Loss_{re}^{soft}$, $0.1*Loss_{re}^{hard}+10*Loss_{re}^{soft}$ and the stage loss function finally adopted in this paper.

In Table 5, compared with $Loss_{re}^{hard}$, $Loss_{re}^{soft}$ by itself was 20.29% higher in the BLUE score, and 11.95% in the ROUGE-L score. The results showed that $Loss_{re}^{hard}$ could not precisely evaluate the quality of candidate answers by using only positive samples to update the network, while $Loss_{re}^{soft}$ could improve the model’s performance by using both positive and negative samples to calculate the gradient; besides, from comparing $0.1*Loss_{re}^{hard}+10*Loss_{re}^{soft}$ with stage-loss function, it could be seen that there are contradictions between the goals of $Loss_{re}^{hard}$ and $Loss_{re}^{soft}$ and that removing $Loss_{re}^{hard}$ can further improve performances.

#### 4.7.2. Different Question Types

The Les MMRC dataset contains six kinds of questions: passage-based, fact-based, opinion-based, and so on. It is challenging for MRC methods to solve complex and abstract question types like passage-based and opinion-based questions. This section discusses performances of IERC-AR on different question types.

In Table 6, the performance of IERC-AR on fact-based questions was better than its performances on the whole dataset, and its performances on number-based and list-based questions were roughly identical with its overall performance. IERC-AR’s ROUGE-L and BLEU scores for definition-based, opinion-based and passage-based questions, were lower than its overall scores. The first three questions in Table 6 mainly focus on questions about what, when, where and who, which are relatively simple and do not require methods that have a powerful reasoning ability. However, the last three are more complex and abstract, which require methods to have an excellent reading comprehension ability, and be able to generate precise context representation by capturing long-range dependencies. 

## 5. Conclusions

This paper proposed an Improved Extraction-based Reading Comprehension methods with Answer Re-ranking (IERC-AR), which can improve performance on Chinese machine reading comprehension tasks. To improve the ability of capturing semantic information at the Chinese word-level, IERC-AR used the contextualized word embedding model built by RoBERTa-WWW to generate precise word representations according to context. Then, to increase the accuracy of answer prediction and enhance the robustness of the model, IERC-AR adopted the candidate answer re-ranking module based on a self-attention mechanism to re-evaluate candidate answers. Furthermore, IERC-AR proposed an end-to-end architecture to integrate the answer extraction module and the answer re-ranking module, which can solve problems of re-encoding and inconsistent data distribution between training and test phases. The experimental results on the Les MMRC dataset showed that IERC-AR not only generated word representation with precise semantic information by fully utilize RoBERTa-WWM based on a bidirectional Transformer, but also increased the accuracy of predicting candidate answers by using an answer re-ranking module based on self-attention. In future work, we plan to introduce external knowledge to improve the process of answering re-ranking.

## Figures and Tables

**Table 1:** The sample in Les MMRC.

**Question**	文章说了什么? (What does the article tell?)
**Question type**	Passage-based question
**Title**	美军首架航母无人机不保证隐身 (The first U.S. aircraft carrier drone does not guarantee stealth.)
**Paragraph**	近日, 美国洛马、波音、通用原子能三家公司分别提出了参加美国海军MQ-25A黄貂鱼舰载无人加油机项目竞标的方案。从三个方案的技术难度上来看, 洛马公司提出的方案采用无尾飞翼构型, 最为复杂……洛马公司的负责人谈起黄貂鱼时表示: 不保证隐身。而且这3款原型机都没有采用诸如锯齿形舱门这类隐身细节设计…… (Recently, three companies including Loma, Boeing and General Atom individually propose schemes about participating in U.S. Navy MQ-25A Stingray Shipborne Unmanned Equipment Project Bidding. From the technical difficulty of three schemes, the solution proposed by Loma uses a non-tail flying wing configuration, the most complicated…… The person in charge of Loma company talked about stingray: not guarantee stealth. And these three original equipment does not adopt such stealth detail designs such as saw tooth cabins.)
**Answer**	美军首架航母无人机不保证隐身 (The first U.S. aircraft carrier drone does not guarantee stealth.)

**Table 2:** Performances of IERC-AR and baselines in the Les MMRC.

	BLEU	ROUGE-L
IERC-AR	**0.8233**	**0.9103**
RoBERTa-WWM based on Fine Tuning	0.7633	0.7491
Match–LSTM	0.7454	0.8880
R-net	0.7965	0.8970
Bi-daf	0.7676	0.8947

**Table 3:** Performances of IERC-AR and IERC-AR without candidate answer re-ranking module in the Les MMRC.

	BLEU	ROUGE-L
IERC-AR	**0.8233**	**0.9103**
IERC-AR without candidate answer re-ranking module	0.8053	0.9056

**Table 4:** Performances of IERC-AR without the answer re-ranking module, its variants and some baselines in the Les MMRC.

	BLEU	ROUGE-L
IERC-AR without candidate answer re-ranking module	**0.8053**	**0.9056**
RoBERTa-WWM based on Fine-Tuning	0.7633	0.7491
M_Reader	0.7882	0.8952
IERC-AR without candidate answer re-ranking module based on BERT-WWM	0.8067	0.9041
IERC-AR without candidate answer re-ranking module based on BERT	0.7503	0.8851

**Table 5:** Performances of IERC-AR with different loss function in the Les MMRC.

	BLEU	ROUGE-L
Stage loss function	**0.8233**	**0.9103**
$Loss_{re}^{hard}$	0.6116	0.7898
$Loss_{re}^{soft}$	0.8145	0.9093
$0.1*Loss_{re}^{hard}+10*Loss_{re}^{soft}$	0.8203	0.9084

**Table 6:** Performances of IERC-AR on different question types in the Les MMRC.

	BLEU	ROUGE-L
fact-based question	0.9328	0.8609
list-based question	0.9181	0.8633
number-based question	0.9032	0.8581
definition-based question	0.8738	0.7485
opinion-based question	0.8668	0.7803
passage-based question	0.8662	0.7170
all question types	0.9181	0.8233

## Data Availability

Data sharing not applicable.
